# Supplementary material for: Licochalcone A Exerts Anti-Cancer Activity by Inhibiting STAT3 in SKOV3 Human Ovarian Cancer Cells
Source: Biomedicines. 2023 Apr 24;11(5):1264. doi: 10.3390/biomedicines11051264 (PMC10215538; doi:10.3390/biomedicines11051264)
Supplement: Supplementary file 1 [file biomedicines-11-01264-s001.zip › biomedicines-2271350-supplementary.pdf]

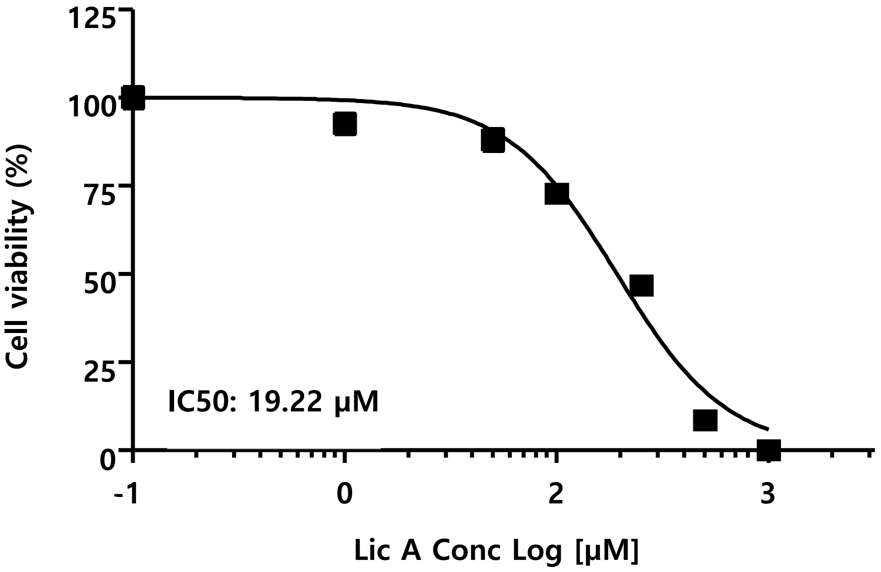

Supplementary Figure S1. Cell viability and corresponding IC 50 values of LicA in SKOV3 cells after incubation for 24 h as determined by CCK-8 assay. All the values are average of replicates expressed relative to cell viability values in control cells normalized to 100%. Data are presented as mean ± SEM.
